# Trajectory of Functional Recovery After Postoperative Delirium in Elective Surgery

**DOI:** 10.1093/geroni/igaf122.1053

**Published:** 2025-12-31

**Authors:** Tammy Hshieh, Jane Saczynski, Edward Marcantonio, Richard Jones, Eva Schmitt, Thomas Travison, Sharon Inouye

**Affiliations:** Brigham and Women’s Hospital, Boston, Massachusetts, United States; Northeastern University, Boston, Massachusetts, United States; Beth Israel Deaconess Medical Center, Boston, Massachusetts, United States; Brown University, Brown University, Rhode Island, United States; Hebrew Rehabilitation Center, Boston, Massachusetts, United States; Hebrew SeniorLife & Harvard Medical School, Hebrew SeniorLife, Massachusetts, United States; Marcus Institute for Aging Research, Hebrew SeniorLife, Harvard Medical School, Boston, Massachusetts, United States

## Abstract

**Objective:**

Describe functional recovery after elective surgery and to determine whether improvements differ among individuals who develop delirium.

**Background:**

Limited studies of older adults have investigated whether delirium influences the trajectory of functional recovery after elective surgery.

**Methods:**

Patients age ≥70 undergoing major elective surgery were assessed daily while in hospital for presence and severity of delirium using the Confusion Assessment Method, and their functional recovery was followed for 18 months thereafter (N = 566). The Activities of Daily Living and Instrumental Activities of Daily Living Scales and the Physical Component Summary of the Short Form-12 were obtained before surgery and at 1, 2, 6, 12, and 18 months. A composite index (standard deviation 10, minimally clinically significant difference 2) derived from these scales was then analyzed using mixed-effects regression.

**Results:**

Mean age was 77 years; 58% of participants were women and 24% developed postoperative delirium. Participants with delirium demonstrated lesser functional recovery than their counterparts without delirium; at 1 month, the covariate-adjusted mean difference on the physical function composite was -1.5 (95% confidence interval [CI] -3.3, -0.2). From 2 to 18 months, the corresponding difference was -1.8 (95% CI -3.2, -0.3), an effect comparable with the minimally clinically significant difference.

**Conclusions:**

Delirium was associated with persistent and clinically meaningful impairment of functional recovery, to 18 months. Use of multifactorial preventive interventions for patients at high risk for delirium and tailored transitional care planning may help to maximize the functional benefits of elective surgery.

